# Development of a model on factors affecting instrumental activities of daily living in people with mild cognitive impairment – a Delphi study

**DOI:** 10.1186/s12883-020-01843-9

**Published:** 2020-07-01

**Authors:** Marina Bruderer-Hofstetter, Sietske A. M. Sikkes, Thomas Münzer, Karin Niedermann

**Affiliations:** 1 School of Helath Professions, Institute of Physiotherapy, Zurich University of Applied Sciences, Winterthur, Switzerland; 2 Department of Health Sciences and Health Policy, University of Lucerne, Lucerne, Switzerland; 3 Alzheimer Center Amsterdam, Amsterdam University Medical Centers / Department of Clinical, Neuro and Developmental Psychology, VU University / Amsterdam Neuroscience, Amsterdam, the Netherlands; 4 Geriatrische Klinik St.Gallen, St.Gallen, Switzerland; 5 Department of Geriatrics and Aging Research, University Hospital and University of Zurich, Zurich, Switzerland

**Keywords:** Instrumental activities of daily living (IADL), Mild cognitive impairment (MCI), Model, Delphi study, Physical function, Cognitive function, Environmental factors, Personal factors

## Abstract

**Introduction:**

The level of function of instrumental activities of daily living (IADL) is crucial for a person’s autonomy. A clear understanding of the nature of IADL and its limitations in people with mild cognitive impairment (MCI) is lacking. Literature suggests numerous possible influencing factors, e.g. cognitive function, but has not considered other domains of human functioning, such as environmental factors. Our aim was to develop a comprehensive model of IADL functioning that depicts the relevant influencing factors.

**Methods:**

We conducted a four-round online Delphi study with a sample of international IADL experts (*N* = 69). In the first round, panelists were asked to mention all possible relevant cognitive and physical function factors, as well as environmental and personal factors, that influence IADL functioning. In the subsequent rounds, panelists rated the relevance of these factors. Consensus was defined as: 1) ≥70% agreement between panelists on a factor, and 2) stability over two successive rounds.

**Results:**

Response rates from the four rounds were high (83 to 100%). In the first round, 229 influencing factors were mentioned, whereof 13 factors reached consensus in the subsequent rounds. These consensual factors were used to build a model of IADL functioning. The final model included: five cognitive function factors (i.e. memory, attention, executive function, and two executive function subdomains -problem solving / reasoning and organization / planning); five physical function factors (i.e. seeing functions, hearing functions, balance, gait / mobility functions and functional mobility functions); two environmental factors (i.e. social network / environment and support of social network / environment); and one personal factor (i.e. education).

**Conclusions:**

This study proposes a comprehensive model of IADL functioning in people with MCI. The results from this Delphi study suggest that IADL functioning is not merely affected by cognitive function factors, but also by physical function factors, environmental factors and personal factors. The multiplicity of factors mentioned in the first round also underlines the individuality of IADL functioning in people with MCI. This model may serve as a basis for future research in IADL functioning in people with MCI.

## Introduction

Instrumental activities of daily living (IADL) are complex tasks, such as managing finances or performing a shopping task [1]. Within the context of cognitive decline, IADLs have been defined as ‘intentional and complex everyday activities for which multiple cognitive processes are necessary, particularly high-level controlled processes’ [2]. Preserved IADL abilities allow people to live independently and to maintain their autonomy. They are crucial on the individual and the societal level [3]. Performance of IADLs are related to an appropriate physical health [4] and cognitive function [3], with IADL limitations being associated with reduced wellbeing [5] and increased caregiver burden, supervision time and total societal costs [6]. Cognitive impairments affect IADL performance [7–9].

Mild cognitive impairment (MCI) is defined as a transient state between normal cognitive ageing and early dementia and is primarily characterized by loss of cognitive function in one or more cognitive domains, but with preserved functional abilities [10]. However, IADL limitations might be present at the MCI state [9, 11, 12]. A recent meta-analysis demonstrated that people with MCI had greater IADL limitations compared to healthy controls, with an effect size of *g* = 0.76 [12]. Furthermore, IADL limitations were found to discriminate people with MCI from people with normal cognition [13] and predict conversion to dementia [14, 15]. These findings may have led to the incorporation of IADL difficulties into the current diagnostic criteria of mild neurocognitive disorder (incorporating MCI) [16].

IADL limitations in people with MCI are associated with cognitive impairment [5, 12]. Empirical data [7] and a meta-analysis [17] estimated that 20 and 23%, respectively, of IADL variability was due to cognition. This implies that other factors also seem to be important in influencing IADL performance. Several studies reported that people with MCI have difficulties in motor function [18–20] and clinical measures, incorporating muscular strength, cardiovascular function and physical activity, predicted a decrease in cognitive function after 1 year [21]. Thus, a clear understanding of the nature of IADL limitations in people with MCI is lacking.

The international classification of functioning, disability and health (ICF) provides a framework for the description of human functioning [22]. The framework considers functioning and disability as outcomes resulting from a health condition, as well as environmental and personal factors and, therefore, may allow a mapping of IADL performance in people with MCI [22]. To date, evidence suggests that there are several physical [18, 20, 23–26] and cognitive function factors [5, 7, 8, 17], as well as personal [27–29] and environmental factors [30–32] influencing IADL performance in people with MCI. However, to our knowledge, previous studies have investigated only a limited number of possible influencing factors, e.g. the association between factors of cognitive function and IADL performance, without [5, 8, 17] or with limited consideration of factors from other domains of the ICF, i.e. factors of physical function, environmental and personal factors [7, 27]. We have, therefore, taken a different approach to modelling the complexity of factors influencing IADL performance. The aim of this study was to develop a model of the physical and cognitive function factors, environmental factors and personal factors contributing to IADL performance in people with MCI, by means of a multiple-round Delphi study based on consensus from an expert panel.

## Methods

### Study design

Between October 2018 and April 2019, a Delphi study was conducted with an international panel of IADL experts (panelists). This design aims to seek consensus of the opinions of a group of panelists through a series of structured questionnaires, i.e. rounds with controlled feedback. Anonymity between panelists is another key element of the study [33]. Two different concepts of consensus were assessed: agreement and stability [34]. Agreement was defined twofold: ≥70% or ≤ 10% of all panelists rate a factor as relevant. Stability determines the consistency of responses and was defined as < 15% difference in percent-agreement between two succeeding rounds [35]. This was used as a measure to stop the Delphi study [36]. The maximum number of rounds was set at four, including the option to omit the fourth round when stability between the second and third rounds was achieved [36].

### Selection of panelists

A selective sampling procedure was used to define the panel [37]. International researchers with authorship of relevant research articles [38] were identified based on a literature search performed in Medline and Web of Science. The search resulted in a total of 163 potential panelists. The panel sample was complemented with researchers from personal networks (SAMS, TM) and, to achieve a broader spectrum in the panel [37], clinicians, neuropsychologists and health professionals who worked with people with MCI on a daily basis in Memory Clinics in the eastern part of Switzerland. The latter were invited by email. Panelists were free to forward the invitation email to whomever they considered as relevant (snowball sampling) [38]. Sixty-nine panelists (*N* = 69) agreed to participate in the Delphi study.

### Procedure

Online questionnaires were pretested and implemented in an EFS (Enterprise Feedback Suite) survey (version 18.3 Questback / Unipark) and distributed by email. In each round, non-responders received a first reminder after 2 weeks and a second reminder 2 weeks later. Questionnaires in subsequent rounds were sent to all panelists who had responded to the questionnaire of the preceding round. Missing data in questionnaires were excluded from data analysis.

### First-round questionnaire and analysis

In the first round, personal details of the panelists (i.e. country of residence, professional background, current occupation / position and years of experience) were collected.

The questionnaire described the aim of the Delphi study, a short summary of current knowledge and the definition of IADL in accordance with Sikkes and Rotrou [2]. The ICF framework [22] was provided as a model for further discussion. The first-round questionnaire asked one open-ended question: *“What are the relevant factors of physical and cognitive function, as well as, personal and environmental factors influencing IADL functioning in people with MCI?”* Panelists were prompted to list all relevant factors for each domain separately (i.e. physical function, cognitive function, environmental factors and personal factors). The first-round questionnaire can be found in the Additional file 1.

A deductive content analysis was performed on all responses [39]: two researchers (MB and a research fellow) independently grouped the mentioned factors into the domains of the ICF framework [22]. Accordingly, environmental factors were defined as factors that are not under the control of the person and personal factors as those possible influencing factors independent of MCI. Depending on personal preference, some factors could be seen both as “personal factors” as well as “environmental factors”, e.g. socio-cultural factors. If appropriate, these factors were included in both domains. Answers describing the same factor in a slightly different manner were merged into one factor [38], whereas specifically-named factors were not comprised into broader functions, e.g. “planning” into “executive function”. Factors were formulated neutrally, without using qualifiers [22], e.g. “impaired vision” was formulated as “seeing function”. Differences in categorization were resolved through discussion with a third researcher (KN) [38].

### Second-round questionnaire and analysis

The questionnaire included all factors mentioned in the first round, together with their frequency, presented for each domain separately. Panelists were then asked to state whether the presented factors were relevant or not.

Percent-agreement on the factors was calculated. Factors reaching ≥70% or ≤ 10% agreement were excluded from the third round questionnaire in accordance with the Delphi methodology [38]. Factors reaching ≥70% percent-agreement were included in the model.

### Third-round questionnaire and analysis

The questionnaire included all factors with a percent-agreement of ≥10% and ≤ 70%, including their frequency and percent-agreement. In addition, a first draft of the model was presented. Panelists were asked to rerate the relevance of these factors. Agreement on the factors was calculated and stability between the second and third rounds was assessed.

### Fourth-round questionnaire and analysis

The second draft of the model was presented. Panelists were asked to provide their feedback on the model and to state whether it was consistent with their conception of IADL performance in people with MCI. Panelists were further asked to rerate on the 10 factors that had not reached consensus or stability in the third round [33].

The feedbacks on the second draft of the model were analyzed using inductive content analysis [39]. Accordingly, one researcher (MB) coded all individual panelists’ responses into categories using a stepwise procedure; frequencies of categories were counted. Percent-agreement on the model was calculated, as well as stability and consensus on the remaining factors. If a factor reached stability and consensus, this factor was included in the final model.

## Results

### Results first round

Sixty panelists (87% response rate; 60 / 69) completed the first-round questionnaire. Panelists (64% female) were from Europe (62%), North and South America (32%) and Australia (6%). Half of the panelists were currently working in academia or research and the other half in the clinical field. Details of professional background and current occupation / position are presented in Table 1. Of all the panelists: 20 (34%) had more than 20 years of experience within their respective field; 15 (25%) between 11 and 20 years; 21 (36%) between five and 10 years; 3 (5%) less than 5 years; one panelist did not provide this information.

**Table 1:** Panel professional background and experience

Professional background	Specialization	n
Physician		17
Psychologist		9
Neuropsychologist		5
Psychopharmacologist		1
Epidemiologist		1
Physical therapist		7
Occupational therapist		9
Nurse		9
Not stated		2
	Geriatrics / Gerontology	6
	Neurology	4
	(Geriatric) Psychiatry	6
	Epidemiology	2
	Anthropology	1
	Research (i.e. PhD)	12
**Current occupation**		
Chair / Dean		3
Professor (assoc. / asst.)		10
Lecturer		4
Researcher		13
Head of department (i.e. memory clinic)		7
Practicing physician		8
Clinical (neuro) - psychologist		10
Dementia specialist		2
Physical therapist		3
Occupational therapist		4
Nursing		7
Professor emeritus / retired		2
Not stated		1

Current occupation multiple naming possible; n = absolute frequency

A total of 229 factors were mentioned in the first round, of which 42 (18%) were physical function factors, 48 (21%) cognitive function factors, 57 (25%) environmental factors and 82 (36%) personal factors, with frequencies ranging from one to 24 (Table 2).

**Table 2:** Mentioned factors

**Physical function factors**									
	First round	Second round	%	Third round	%	% diff.	Fourth round	%	% diff.
^a^vision / seeing functions	13	39	75						
vision acuity	4	6	11.5	6	11.8	0.2			
eye movement functions	1	0							
^a^hearing functions	18	37	71.2						
sensory functions	2	7	13.5	7	13.7	0.3			
proprioceptive functions	1	6	11.5	5	9.8	−1.7			
touch functions	3	6	11.5	3	5.88	−5.7			
smell functions	1	1	1.9						
pain	5	19	36.5	24	47.1	10.5			
vestibular functions	3	8	15.4	8	15.7	0.3			
vestibular function of balance	1	2	3.8						
stability	2	3	5.8						
^a^balance	20	37	71.2						
^a^mobility / gait functions	17	38	73.1						
fall risk / fall experience	2	10	19.2	16	31.4	12.1			
walking speed	3	4	7.7						
^c^functional mobility (e.g. stair climbing)	4	22	42.3	32	62.7	20.4	31	79.5	−16.7
ability to travel	3	4	7.7						
general physical endurance functions	16	26	50	19	37.3	−12.7			
aerobic capacity	1	1	1.9						
fatigability	3	10	19.2	7	13.7	−5.5			
muscle power functions (general physical strength)	15	30	57.7	33	64.7	7			
lower limb power (lower extremity strength)	4	2	3.8						
grip strength	5	7	13.5	11	21.6	8.1			
upper extremity strength	1	1	1.9						
manual dexterity (fine motor skills)	9	25	48.1	27	52.9	4.9			
fine motor coordination	5	12	23.1	15	29.4	6.3			
coordination	4	9	17.3	11	21.6	4.3			
control of body movement functions	1	2	3.8						
visuo-motor coordination capacity	1	8	15.4	16	31.4	16	13	33.3	2.0
tremor	1	0							
mobility of joints functions (e.g. range of motion)	13	18	34.6	8	15.7	−18.9	6	15.4	−0.3
mobility of the spine and cervical spine	1	0							
gross motor function	3	6	11.5	3	5.88	−5.7			
motor speed	5	11	21.2	14	27.5	6.3			
agility	1	0							
functional reach	1	4	7.7						
functions of the cardiovascular system	2	5	9.6						
cardiorespiratory reserve	1	1	1.9						
blood pressure	1	1	1.9						
cholesterol values	1	1	1.9						
Respiratory functions	2	2	3.8						
**Cognitive function factors**									
	First round	Second round	%	Third round	%	% diff.	Fourth round	%	% diff.
^a^attention functions	23	39	78						
sustaining attention	9	2	4						
shifting attention	5	4	8						
dividing attention	6	7	14	10	19.6	5.6			
sharing attention	2	2	4						
processing speed functions	7	19	38	17	33.3	−4.7			
reaction time	2	4	8						
^a^executive functions	24	39	78						
sequencing	4	2	4						
^b^organization and planning	11	27	54	37	72.5	18.5			
cognitive / mental flexibility	7	22	44	22	43.1	−0.9			
insight	9	2	4						
judgement / decision making	5	14	28	22	43.1	15.1	25	64.1	21.0
^b^problem solving / reasoning	8	27	54	36	70.6	16.6			
inhibition	3	2	4						
initiation	1	1	2						
^a^memory functions	25	42	84						
learning	4	3	6						
short-term memory	5	3	6						
long-term memory	3	0							
episodic memory	3	2	4						
semantic memory	1	0							
working memory	7	9	18	18	35.3	17.3	24	61.5	26.2
prospective memory	2	3	6						
retrieval and processing of memory	7	4	8						
language functions	13	33	66	25	49.0	−17	26	66.7	17.6
language comprehension (written and spoken)	7	11	22	10	19.6	−2.4			
semantic fluency	4	1	2						
semantic knowledge	1	0							
language execution	3	1	2						
word finding	3	3	6						
calculation functions	9	4	8						
abstraction	1	0							
perceptual functions	8	12	24	7	13.7	−10.3			
perceptual-motor functions	1	1	2						
visuo-spatial functions	9	21	42	22	43.1	1.1			
visuo-perceptual functions	5	2	4						
psychomotor functions	5	6	12	6	11.8	−0.2			
orientation	5	11	22	17	33.3	11.3			
energy and drive / stamina	2	3	6						
metacognition	3	3	6						
motivation	16	29	58	28	54.9	−3.1			
mood	9	12	24	13	25.5	1.5			
alertness / vigilance	5	7	14	12	23.5	9.5			
awareness	1	3	6						
intelligence	3	1	2						
social cognition	8	15	30	15	29.4	−0.6			
emotional functions	8	13	26	10	19.6	−6.4			
**Environmental factors**									
	First round	Second round	%	Third round	%	% diff.	Fourth round	%	% diff.
parental beliefs	2	0	0						
societal attitudes	4	6	12	3	5.9	−6.1			
social expectations	3	5	10	3	5.9	−4.1			
social norms	4	11	22	3	5.9	−16.1	4	10.3	4.4
socio-cultural factors	4	19	38	22	43.1	5.1			
widowed / changes in personal network	3	7	14	7	13.7	−0.3			
family support	3	11	22	16	31.4	9.4			
^a^social network / social environment	13	35	70						
^c^network / social support	11	16	32	31	60.8	28.8	29	74.4	13.6
loneliness / isolation	5	20	40	25	49.0	9			
personal assistance available	3	4	8						
immediate family (e.g. children, siblings)	6	9	18	8	15.7	−2.3			
extended family (e.g. spouse)	4	6	12	3	5.9	−6.1			
weather	1	1	2						
climate	3	0							
extreme temperatures	1	0							
noise	3	3	6						
adequate light	1	2	4						
air quality / pollution	3	1	2						
place of residence (rural versus urban environment)	7	31	62	25	49.0	−13			
neighborhood	4	7	14	9	17.6	3.6			
age-friendliness of environment	1	8	16	11	21.6	5.6			
environmental demands	3	6	12	3	5.9	−6.1			
familiarity with environment	1	7	14	9	17.6	3.6			
challenging environment	3	0							
physical environment / living environment	6	0							
presence of gangs	1	0							
type of house / apartment	3	3	6						
adaptation / age-friendliness / safety of home environment	7	13	26	10	19.6	−6.4			
Housing / immediate home environment	9	19	38	21	41.2	3.2			
accessibility of the house / apartment	1	4	8						
living form	5	6	12	5	9.8	−2.2			
living with family / family nearby	3	5	10	7	13.7	3.7			
living situation (independent / dependent)	1	5	10	8	15.7	5.7			
financial situation / resources	13	32	64	21	41.2	−22.8	17	43.6	2.4
financial resources for dental care	1	0							
living condition	1	0							
access to ICT	4	4	8						
products and technology for personal use in daily living	2	6	12	10	19.6	7.6			
communication technology	2	1	2						
personal devices (apps)	1	0							
technological aids / means	2	2	4						
means for physical impairments / access to assistive devices	6	16	32	14	27.5	−4.5			
mobility aids	2	1	2						
access to cognitive protheses	1	2	4						
access to information and use of different channels	2	1	2						
quality of instructions (easy to understand for MCI)	1	0							
access to and dependence on transportation	3	8	16	9	17.6	1.6			
accessibility / distance to public transport	6	16	32	15	29.4	−2.6			
accessibility / distance to (social) activities	4	10	20	9	17.6	−2.4			
accessibility / distance to facilities	7	12	24	14	27.5	3.5			
country of residence	1	5	10	2	3.9	−6.1			
insurance policy of a country	1	1	2						
official structured support / possibilities (e.g home care)	4	11	22	10	19.6	−2.4			
educational opportunity	1	2	4						
availability and access to health care	3	12	24	12	23.5	−0.5			
policy	1	2	4						
**Personal factors**									
	First round	Second round	%	Third round	%	% diff.	Fourth round	%	% diff.
age	8	26	52	23	45.1	−6.9			
sex / gender	6	13	26	11	21.6	−4.4			
race	1	0							
^a^education	15	35	70						
professional background	2	6	12	6	11.8	−0.2			
professional occupation	3	2	4						
socio economic status	7	24	48	20	39.2	−8.8			
genetics (e.g. predisposition)	4	6	12						
body composition	2	2	4						
body mass index	1	2	4						
weight / obesity	2	1	2						
(physical) condition / fitness	8	25	50	28	54.9	4.9			
predetermined physical capacity	1	0							
cognitive health	1	10	20	14	27.5	7.5			
cognitive habits	4	7	14	6	11.8	−2.2			
cognitive reserve	2	9	18	10	19.6	1.6			
nutrition / liquid intake	4	7	14	6	11.8	−2.2			
nutritional state	2	2	4						
vitamin / vitamin deficiency	1	0							
sleep quality	2	12	24	12	23.5	−0.5			
circadian rhythm	2	1	2						
balance between recreation and activity	1	3	6						
values	2	5	10	3	5.9	−4.1			
beliefs	3	4	8						
religion / spirituality	5	5	10	2	3.9	−6.1			
personal attitudes	1	1	2						
self-concept	3	2	4						
self-esteem	2	2	4						
self-satisfaction	1	0							
self-efficacy	4	13	26	16	31.4	5.4			
perceived stress	2	2	4						
well-being	1	0							
sense of purpose in IADL tasks	2	10	20	16	31.4	11.4			
personality	10	22	44	20	39.2	−4.8			
social skills	7	8	16	10	19.6	3.6			
conation	2	0							
desire for independence	2	5	10	9	17.6	7.6			
behavior pattern	3	0							
general initiative-taking	1	2	4						
extraversion	2	0							
being open (e.g. willing to learn new things)	3	5	10	3	5.9	−4.1			
coping strategies	8	25	50	28	54.9	4.9			
frustration tolerance	1	0							
willing to ask for and accept someone’s help	3	8	16	13	25.5	9.5			
flexibility / creativity	2	1	2						
resilience	3	8	16	5	9.8	−6.2			
hobbies	2	1	2						
interests	4	5	10	8	15.7	5.7			
maintenance of habits (e.g. hobbies, interests, sexuality)	1	6	12	8	15.7	3.7			
personal hygiene	3	0							
personal habits (e.g. not have done certain IADL lifelong)	1	8	16	13	25.5	9.5			
personal routine	3	3	6						
personal (daily) structure	2	2	4						
physical activity (past and current)	6	15	30	15	29.4	−0.6			
enjoy of physical activity	2	0							
social activities	2	3	6						
moral conduct	3	0							
family position	2	1	2						
(gender) roles	4	0							
social integration / connectedness	8	15	30	18	35.3	5.3			
socio-cultural background	13	10	20	10	19.6	−0.4			
upbringing	3	0							
literacy / health literacy	1	3	6						
experience / biography	7	5	10	4	7.8	−2.2			
experience - (e.g. familiarity with certain IADL tasks)	6	10	20	21	41.2	21.2	23	59	17.8
computer literacy	3	0							
physical health	6	7	14	8	15.7	1.7			
neurological medical conditions	3	4	8						
musculoskeletal medical conditions	5	3	6						
treatment of physical illness	1	0							
multimorbidity	1	3	6						
comorbidities	3	8	16	12	23.5	7.5			
disease duration	1	0							
frailty	5	6	12	12	23.5	11.5			
psychological health	8	17	34	23	45.1	11.1			
psychosis	2	0							
depression	13	17	34	20	39.2	5.2			
anxiety	9	4	8						
current medication / possible side effects	5	3	6						
smoking	3	1	2						
alcohol consumption	2	2	4						
addiction / substance misuse	5	2	4						

Factors mentioned in the first round, frequency, %: percent-agreement between panelists; %-diff: difference in percent-agreement between two succeeding rounds – a positive number indicates more agreement

^a^factors included after second round

^b^factors included after third round

^c^factors included after fourth round

### Results second round

Fifty-three panelists (88% response rate; 53 / 60) completed the second-round questionnaire. One questionnaire was excluded in data analysis due to missing data, in two questionnaires data was missing for cognitive function, environmental and personal factors and in one questionnaire data was missing for environmental and personal factors. The panel reached consensus on 126 factors (55%). Nine of these factors were rated as relevant by ≥70% of panelists and were included in the model, whilst 117 (51%) factors were rated as relevant by ≤10% and were subsequently excluded from the third round (Table 2). Overall, 103 (45%) factors did not reach consensus in the second round and were included in the third-round questionnaire (Table 2).

### Results third round

Fifty-three panelists (100% response rate; 53 / 53) completed the third-round questionnaire. Two questionnaires were excluded from data analysis due to missing data. Of the remaining 103 factors, two (2%) reached consensus and were included in the model. Stability of responses between the second and third round was ascertained for 93 (90%) factors. Ten factors (10%) did not reach stability and were therefore included in the fourth-round questionnaire.

### Results fourth round

Forty-four panelists (83% response rate; 44 / 53) responded to the fourth-round questionnaire. Thirty-three (62%; 33 / 53) panelists provided feedback on the model with 28 (85%) stating that the model met their conception of IADL functioning in people with MCI. Feedback on the model covered: factors not included in the model; lack of weighting and relatedness of the factors; one panelist questioned the method itself (Table 3).

**Table 3:** Critical comments on the model

^**a**^Categories	Frequency (percent)
Same weighting for all factors	4 (12%)
Balance as separate factor from mobility	4 (12%)
Mental health not included	4 (12%)
Relatedness of factors not included	3 (9%)
Problem solving / planning included in executive functions	3 (9%)
Environmental factors - products and technology not included	3 (9%)
Environmental factors - natural environment not included	2 (6%)
Vision / hearing functions not gathered as sensory functions	2 (6%)
Physical function factors (others than balance) not included	2 (6%)
Visuospatial functions not included	1 (3%)
Language functions not included	1 (3%)
Fine motor skills not included	1 (3%)
Motivation not included	1 (3%)
Method not appropriate	1 (3%)

^a^Categories based on qualitative content analysis from the feedback provided on the model

**Fig. 1:**
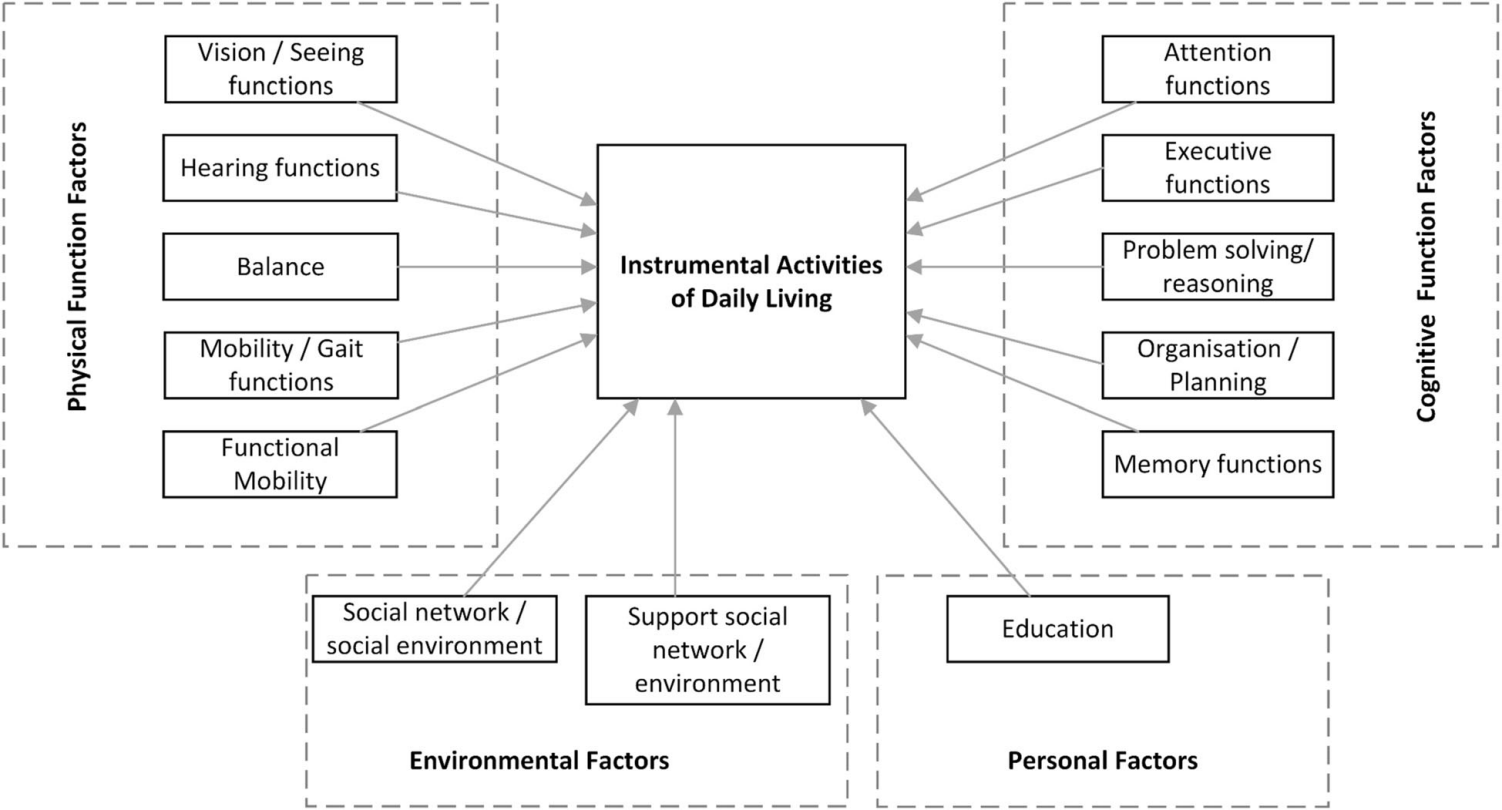
Model of IADL functioning in people with MCI

The two additional factors reached consensus and stability between the third and fourth rounds and were consequently included in the final model (Fig. 1). Stability between the third and fourth rounds was not reached for five factors (50%), i.e. judgment / decision making, working memory, language functions, financial situation and experience / familiarity with certain IADL tasks (Table 2).

## Discussion

The results of this Delphi study illustrate how panelists from the academic / research and clinical practice perspectives agreed on several factors of cognitive and physical functions, as well as personal factors and environmental factors, that are thought to influence IADL performance in people with MCI.

To our knowledge, this study is the first to propose a comprehensive model on the influencing factors on IADL performance in people with MCI, incorporating all domains of the ICF framework. IADL performance in people with MCI is highly individual and might be dependent on the culture and environment a person lives in, which was represented by the wide variety of factors mentioned in the first round of the Delphi survey. Although the Delphi method included relevant researchers and clinicians, the model may not be conclusive. However, a substantial number of the factors reaching consensus are consistent with the findings from empirical data [17–20, 24–29, 40, 41], while others have been neglected in the literature so far, e.g. functional mobility. Thus, our model might provide a better understanding of IADL functioning in people with MCI and serve as a ground for future research. Cross-sectional or cohort studies on IADL functioning might use the model as a base to decide which factors should be investigated; intervention studies might use our model as theoretical background in the development of novel interventions that aim to improve IADL functioning in people with MCI. Nonetheless, our model might have implications for clinical practice by strengthening the awareness that IADL functioning is influenced not merely by cognition. Considering all factors in the treatment of people with MCI with IADL impairments might help to improve their level of functioning; for instance, by counteracting impaired sensory functions with an appropriate aid. Additionally, our model might have an impact on the way IADL functioning is assessed.

### Cognitive function factors

Multiple cognitive function factors were included in the model. Consensus was reached for memory, attention and executive function, as well as executive function subdomains organization / planning and problem solving / reasoning. The bulk of literature investigating the question of which cognitive domains account for IADL performance is not consistent. Despite the widely accepted assumption that IADL performance is mainly affected by cognition, Royall et al. suggest that, based on empirical data, less than 8% of IADL variance is explained by cognition [42]. Furthermore, in another study, the same group ascertained in their empirically- based model that intelligence accounts for at least 50% of the variance in IADL performance in people with MCI [43]. The fact that IADL performance is independent of cognitive performance measures and the fraction of intelligence is related to IADL, may both serve as a dementia severity metric [43]. However, in our study intelligence did not reach consensus and our results contradict the findings of empirical studies. On the other hand, in their meta-analysis, McAlister et al. revealed that cognitive functions accounted for 23% of the variability in IADL performance in people with MCI [17]. Among the cognitive domains, executive function (37%), attention (33%) and memory (23%) explained a certain amount of variance in IADL performance, while planning / organization and problem solving / reasoning explained a smaller amount of variance [17]. In our study, the subdomains planning / organization and problem solving / reasoning were explicitly raised by the panel and reached consensus in addition to executive function. One may argue, that the subdomains are already encompassed by executive function. However, executive function is an umbrella term [44] and heterogeneous definitions of subdomains are found in the literature [17, 45]. Therefore, we did not exclude the subdomains from the model. Furthermore, an exclusion of these subdomains would have contradicted our predefined cut-offs regarding consensus. The meta-analysis by McAlister et al. also detected other cognitive domains and executive function subdomains not included or explicitly mentioned in our model (e.g. switching, judgment / decision making and working memory) that explained a remarkable amount of variance [17]. Therefore, factors that did not reach consensus in our Delphi process, but that showed a remarkable amount of agreement, might still be added to the model in future studies, e.g. language functions (67%).

### Physical function factors

Certain IADL tasks need appropriate sensory functions. Not surprisingly, visual and hearing functions were included in the model in accordance with the current literature. A longitudinal study indicated that visual and hearing impairments are related to self-reported functional impairments in old people [24]. Furthermore, sensory restrictions are associated with slight IADL changes [27] and the presence of visual and hearing impairment in combination with cognitive decline was associated with impaired IADL performance in older adults [25].

Balance was included in the model even though some panelists suggested that balance is a subdomain of gait functions. Impaired balance does have an impact on gait function, but several IADL tasks also require static balance abilities [46]. For this reason, balance was not summarized under gait functions. Literature on balance in people with MCI is sparse. However, studies using instrumented assessments did find impaired balance functions in people with MCI [20]. Moreover, studies using clinical assessments of balance, e.g. POMA, revealed an association between IADL performance and balance in people with MCI [26].

Mobility / gait functions were included in the model, which is supported by the current literature [20]. Different aspects of gait function were found to be impaired in people with MCI [19, 47–49]. A remarkable number of IADLs require sound gait functions, e.g. doing the shopping or using public transport.

A further factor included in the model was functional mobility, e.g. walking stairs or functional reach, although functional mobility related to IADL performance has had little attention in literature to date. Therefore, future studies investigating IADL performance in people with MCI should consider functional mobility as a possible influencing factor.

Physical function factors that might affect IADL performance [40], e.g. muscle power functions, reached a remarkable percent-agreement (65%), but insufficient consensus to be included in the model. Mobility/gait functions and functional mobility presume, inter alia, appropriate muscle power functions. In addition, grip strength may be associated with functional impairments in people with MCI [23]. Therefore, the factor muscle power functions might be worth considering in studies investigating the influence of physical function factors on IADL performance in people with MCI.

### Environmental factors

Based on the panelists’ suggestions, “Network / Social Environment” and “Network / Social Environment Support” were included in the model. Intervention studies including study partners reported positive findings on IADL performance in people with MCI [31, 32], leading to the conclusion that these factors play an important role.

Several environmental factors were mentioned in the first round but failed to reach consensus. Some were also raised during the feedback on the model: natural environment, e.g. place of residence, housing and products and technology (technical aids). The importance of compensatory strategies and use of technical aids in the performance of IADL in people with MCI has been highlighted in literature [30]. Furthermore, these factors underline the individuality of IADL functioning and might be considered in the design of future studies or interventions on IADL performance in people with MCI.

### Personal factors

The only personal factor included in the model was education. Education and cognitive function might be related in people with MCI. Education is usually included as a possible confounder in empirical studies. However, conclusions from literature are not clear. In a longitudinal study on a sample of Asian older adults, lower education was associated with greater IADL dependence [28], while a higher level of cognitive reserve delayed the onset of cognitive decline [29]. In contrast, a meta-analysis did not find education as a mediator of the relationship between cognitive function and IADL [17].

Literature suggests additional personal factors that might influence IADL performance in MCI [3] but with inconsistent findings. Age was found to be associated with impaired IADL performance in MCI [28, 50], as well as depression [28, 51], frailty [26], physical activity [52] and comorbidities [28, 53]. In contrast, Mariani and colleagues revealed that IADL performance was more strongly related to cognitive function than physical comorbidities [27]. The inconclusive findings in literature, as well as the ratings in the Delphi process, underline the individual nature of IADL performance in people with MCI.

### Strength and limitations

One strength of our study is the number of panelists, with half of them working in research and academia and the other half in clinical settings. The great amount of experience of the panelists in the field of MCI and IADL performance is also noteworthy. Unfortunately, the panel did not include experts from the Asian or African continents due to non-response and we consequently do not know if and how African or Asian panelists would have influenced the model. The response rates in the first three rounds of the Delphi survey were very high. Another strength of this study is that it used a different approach to modelling IADL functioning in people with MCI and the new insights could provide a basis for future research.

This study also has several limitations. A Delphi study reports only the results from a consensus of expert opinions on a topic and could contradict findings from empirical studies [38]. Performing a systematic review would have been a different approach to investigate the possible contributing factors on IADL performance in MCI. However, systematic reviews performed in this field have faced similar problems: the constructs of interest (i.e. MCI, IADL) have been defined and operationalized in different ways [5, 7, 12, 17, 54]. Furthermore, the type and number of assessments used to measure the outcomes of interest were heterogeneous [5, 7, 12, 17, 54]. Moreover, the results are limited to the factors investigated in the included studies and might not be fully encompassing. Comparable problems would arise from empirical studies: a retrospective analysis of pre-existing data sets would be limited to the outcomes assessed; in a prospective design, it remains unclear which factors should be assessed, given the huge range of possibilities, e.g. domains provided by the ICF. Thus, we took a deductive approach to build a model (theoretical) based on the panelists great insight and understanding of IADL functioning in people with MCI; we suggest our model should be used as a starting point for further elaboration based on an inductive approach using empirical data.

The definition of consensus in Delphi studies is somewhat arbitrary [34]. One might argue, that the predefined cut-off level of ≥70% percent-agreement for factors to be included in the model was set too low. However, this study included a heterogeneous sample of panelists and, therefore, very high percent-agreements were not anticipated. Alternatively, the cut-off might have been set too high resulting in relevant factors with substantial percent-agreement being excluded from the model. These might be considered in future studies, as previously discussed.

Finally, due to the design of our study, it was not possible to weight the factors. In the feedback round some panelists pointed out that some factors are more important than others. Therefore, weighting of the factors in general and across different cultures should be incorporated in future studies investigating IADL performance in people with MCI.

## Conclusion

The results of this study suggest that IADL performance in people with MCI is affected not only by cognitive function factors, but also by various physical function factors, personal factors and environmental factors. Therefore, it is crucial to consider all these factors in future studies in people with MCI exploring IADL performance, as well as in the design and investigation of new interventions to improve everyday activities. Finally, our results may have implications for clinical practice in people with MCI, both in the methods of assessing IADLs and the treatment of IADL impairments.

## Supplementary information

**Additional file 1.** First-round Questionnaire. The file contains the questionnaire from the first round; it was downloaded from EFS survey on 23. September 2019: https://ww2.unipark.de/www/print_survey.php?syid=515897&menu_node=print2.

## Data Availability

The datasets generated and analyzed during the study are available upon reasonable request to the corresponding author.

## Abbreviations

IADL: Instrumental Activities of Daily Living
ICF: International Classification of Functioning, Disability and Health
MCI: Mild Cognitive Impairment
POMA: Performance Oriented Mobility Assessment
