# Supplementary material for: Development of a model on factors affecting instrumental activities of daily living in people with mild cognitive impairment – a Delphi study
Source: BMC Neurol. 2020 Jul 1;20:264. doi: 10.1186/s12883-020-01843-9 (PMC7329426; doi:10.1186/s12883-020-01843-9)
Supplement: Supplementary file 1 — Additional file 1. First-round Questionnaire. The file contains the questionnaire from the first round; it was downloaded from EFS survey on 23. September 2019: https://ww2.unipark.de/www/print_survey.php?syid=515897&menu_node=print2. [file 12883_2020_1843_MOESM1_ESM.pdf]

Additional file 1 – Questionnaire of the first round

1 Welcome

Thank you very much for participating in our study and filling out the questionnaire. Some background information on the Delphi survey are provided below.

This is the first round of the international Delphi Survey aiming at developing a theoretical framework (model) on the contributing aspects of physical capacity and cognitive function as well as environmental and personal factors on instrumental activities of daily living (IADL) in people with mild cognitive impairment (MCI). Thus, we intend to define what factors influence IADL functioning in people with MCI and whether and how they are interrelated.

Impairment in IADL is apparent to some extent at the MCI stage. This is also reflected by the incorporation of IADL difficulties into current diagnostic criteria (e.g. DSM V). However, a clear understanding of the nature of this disability is lacking. Cognition and IADL are somehow interrelated, but a recent Meta-Analysis found that a large amount of variance in IADL performance remained unexplained by cognition and that some subdomains are more correlated to IADL than others (McAlister et al. 2016). Therefore, other factors such as motor functions, behavioral problems and visual problems may play an important role (Royall et al. 2007).

Two starting points:

1. The model from the international classification of functioning, disability and health: in which physical capacity (e.g. walking abilities, pure motor function) and cognitive function (e.g. global cognition, subdomains) represent 'function'; IADL is the 'activity'; personal factors are for example education and living situation and environmental factors are for example use of notes and technical aids.

2. The definition of IADL proposed by Sikkes & de Rotrou (2014): IADL are intentional and complex everyday activities for which multiple cognitive processes are necessary, particularly high-level controlled processes.

On the next page, some personal details will be asked. On the consecutive page, we will ask you one open ended question.

Personal Details

What is your professional background?

What is your current occupational activity?

How many years of experience do you have within the field of people with MCI and / or IADL functioning?

Please choose

- < 5 years ☐
- 5 to 10 years ☐
- 11 to 15 years ☐
- 16 to 20 years ☐
- > 20 years ☐

What is your country of residence?

What are the relevant influencing factors of physical capacity, cognitive function, personal and environmental factors on IADL functioning in people with MCI?

Please provide all relevant physical capacity factors.
List all physical capacity factors that come to your mind. The order is not important.

Please provide all relevant cognitive function factors.
List all cognitive function factors that come to your mind. The order is not important.

Please provide all relevant personal factors.
List all personal factors that come to your mind. The order is not important.

Please provide all relevant environmental factors.
List all environmental factors that come to your mind. The order is not important.

Thank you for filling out the questionnaire.

We are going to analyze all responses as soon as possible and will fed them back to you in the second round. In the second round, we will ask you to rate on the relevance of all factors provided by the panel and to state their relatedness.

You will receive the invitation for the second round at the latest in mid November.

CLOSE WINDOW
